# Spheres-in-Grating Assemblies with Altered Photoluminescence and Wetting Properties

**DOI:** 10.3390/nano12071084

**Published:** 2022-03-25

**Authors:** Iuliana M. Handrea-Dragan, Adriana Vulpoi, Cosmin Farcău, Ioan Botiz

**Affiliations:** 1 Institute for Interdisciplinary Research in Bio-Nano-Sciences, Babes-Bolyai University, 400271 Cluj-Napoca, Romania; iuliana.dragan@ubbcluj.ro (I.M.H.-D.); adriana.vulpoi@ubbcluj.ro (A.V.); cfarcau@itim-cj.ro (C.F.); 2 Faculty of Physics, Babes-Bolyai University, 400084 Cluj-Napoca, Romania; 3 National Institute for Research and Development of Isotopic and Molecular Technologies, 400293 Cluj-Napoca, Romania

**Keywords:** polymer embossing, groove rectangular grating, fluorescent spheres, convective self-assembly, spheres-in-grating assemblies, photoluminescence properties

## Abstract

In this work, we report the fabrication of spheres-in-grating assemblies consisting of equally spaced parallel rectangular grooves filled with fluorescent spheres, by employing embossing and convective self-assembly methods. The developed hierarchical assemblies, when compared to spheres spin-cast on glass, exhibited a blueshift in the photoluminescence spectra, as well as changes in wetting properties induced not only by the patterning process, but also by the nature and size of the utilized spheres. While the patterning process led to increased hydrophobicity, the utilization of spheres with larger diameter improved the hydrophilicity of the fabricated assemblies. Finally, by aiming at the future integration of the spheres-in-grating assemblies as critical components in different technological and medical applications, we report a successful encapsulation of the incorporated spheres within the grating with a top layer of a functional polymer.

## 1. Introduction

Today, a variety of miniaturized optoelectronic, photonic, and sensorial devices, as well as other critical components for biomimetics or medical utility [1,2,3,4,5,6], are being developed and improved through the use of simple fabrication methods based on lithographic processes [2,5], electrohydrodynamic spray deposition [1], electrophoretic deposition [3], and others [4,5,6], with the final goal to boost and advance the fields of energy, detection, optoelectronics, and photonics [2,7,8,9], as well as to develop specific medical applications [10,11,12,13,14,15,16]. The efficiency of most of such devices and components strongly relies on delicate (periodic) surface relief structures of specific dimensions, shape, or function [17,18,19,20,21,22,23,24,25,26,27], which can mainly be produced by employing top–down and bottom–up patterning technologies [17,28,29,30,31], or by combining the two categories [5,32,33].

Among the multitude of patterning techniques that include photolithography, electron-, proton-, and ion-beam lithography, scanning lithography, particle lithography, and stencil lithography [5], nanoimprint lithography has become a dominant tool to fabricate micro- and nanoscale periodic surface relief patterns at low cost through the employment of soft, elastomeric stamps and molds [34,35,36]. Due to its simplicity and proven efficiency in producing high-quality nanoscale surface relief structures [37,38,39,40,41,42], thermal nanoimprint lithography (e.g., embossing) is an ideal method to rapidly create several-centimeter-large polymeric surface relief structures, including gratings consisting of parallel rectangular grooves. Such size of the patterned samples allows embossing to be efficiently coupled with convective self-assembly (CSA; a technique developed in the field of materials and colloidal chemistry [43,44,45], and predominantly used to deposit colloidal solutions onto centimeter-scale solid substrates [46,47]), in order to incorporate spheres of specific dimensions and function into various surface relief structures and, thus, to create ordered/disordered hierarchical platforms. The latter are highly desirable components in a plethora of current technological applications described in detail elsewhere [5].

Herein, we present the fabrication of spheres-in-grating assemblies exhibiting altered emissive properties through the employment of cost-effective patterning–filling methodologies. All of the functional assemblies were prepared from spin-cast polystyrene (PS) films that were patterned using the embossing method, followed by deposition—via adapted CSA—of various fluorescent spheres into their periodic groove-like patterns. The obtained hierarchical platforms were characterized by means of UV–Vis and photoluminescence (PL) spectroscopy, as well as optical and scanning electron microscopy (SEM). The fluorophores used in staining the polymeric spheres can be used in bioimaging applications, as fluorescent tracers, or for covalent coupling of proteins in biomedical studies.

## 2. Materials and Methods

Atactic polystyrene (PS) with a molecular weight of approximately 192 kDa was purchased from Sigma-Aldrich (Schnelldorf, Germany). PS solution was prepared by dissolving 200 mg of PS in 1 mL of toluene (a viscous, yet highly homogeneous PS solution was obtained after keeping it for 48 h at room temperature), and was then further used, by spin-casting (2000 rpm for 30 s using a LAURELL 650M spin-coater), ~ to create 4 µm thick PS films on a UV–ozone-cleaned glass substrate.

The embossing of PS films was conducted at 180 °C. Polydimethylsiloxane (PDMS) molds were placed on PS films and uniformly pressed by a weight of 1 kg for 4 min, to ensure that the desired pattern was being efficiently transferred. The patterns on the PDMS molds consisted of parallel grooves featuring a lateral periodicity of 4 μm (each groove of a width of 2.5 μm was separated by a wall of 1.5 μm; the depth of the grooves was 2 μm). The resulting PS gratings, consisting of high-quality periodic rectangular grooves, were treated in UV–ozone for 20 min prior to any other use.

The grooves were further filled with various spheres through the use of CSA. Fluorescent spheres of different diameters and colors were acquired from SPHERO^TM^ (Lake Forest, IL, USA) as dilute suspensions of PS beads stained with various organic fluorescent dyes (details on spheres are available in Table S1 in the Supplementary Materials). The homemade CSA tool was comprised of a motorized translation stage, based on a linear actuator from Zaber Technologies—Vancouver, BC, Canada, which is capable of moving at speeds ranging between ~4.7 μm/s and 8 mm/s. A cover glass that acted as a blade was fixed in the near vicinity of the substrate at the desired angle, while the colloidal suspension was placed on the substrate, underneath and nearby the edge of the blade. Translation was always executed along the direction parallel to the grooves. To obtain an efficient filling of the gratings with the spheres, parameters such as translation speed, sample temperature, concentration of fluorescent spheres in the colloidal suspension, etc., were optimized and used (see further details elsewhere [46,48]).

The quality of the PS gratings as well as of the gratings filled with specific spheres was assessed by optical and scanning electron microscopy (SEM). Optical images were taken with a KERN Microscope operating in reflection mode, to ensure the overall quality of our samples over larger areas. SEM images were obtained by using a FEI Quanta 3D microscope equipped with an EDT detector, operating in high vacuum mode, using an acceleration voltage of 10 kV. Fluorescence images of the spheres-in-grating assemblies were recorded by using a 532 nm excitation line on a WITec Alpha300R Raman microscope in a confocal scanning configuration. Both excitation and collection of the emitted light were achieved using a 100 × /0.9 NA objective. Light was then passed to the spectrometer through an optical fiber with a 25 μm diameter entrance hole, acting as a confocal pinhole. 20 × 20 μm^2^ sample areas were scanned with a resolution of 128 × 128 pixels. A relatively high scanning speed (i.e., short integration time, 20 µs/pixel) was used in order to avoid fluorophore photodegradation during the scan. To further analyze the optical properties of the created structures, we recorded the absorption and emission spectra. The absorption spectra were recorded with a 1 nm spectral resolution double-beam UV–Vis–NIR spectrophotometer (Jasco V-670, Tokyo, Japan). Photoluminescence spectra were recorded at room temperature on an FP-6500 spectrofluorometer from JASCO (excitation wavelength range of 220–750 nm).

A contact angle goniometer (Ossila Ltd., Sheffield, UK) was used to characterize the wettability of the fabricated surfaces. The static contact angle of water was measured by dispensing 5 μL drops of deionized water on the samples, recording images, and analyzing these images using the software provided by Ossila (note that the images were taken along the axis perpendicular to the grooves’ direction). The measurement was repeated on several random positions across the surface to obtain average contact angle values.

For the encapsulating strategy, ~3 µm thick and homogeneous poly(vinylidene fluoride-co-trifluoroethylene)/P(VDF-TrFE) films were spin-cast (at 500 rpm for 60 s) from acetonitrile-based solutions (15 wt%; previously stirred overnight on a hot plate at 70 °C) on glass substrates that were not treated in the UV–ozone, in order to ensure an easy detachment of the resulting polymer film. P(VDF-TrFE) films were then manually separated from the glass substrate and used to coat the spheres-in-grating assemblies. To ensure a good contact between the encapsulating film and the gratings, a stream of heat produced by a hair drier was applied for 30 s over the final device.

## 3. Results and Discussion

**Figure 1:**
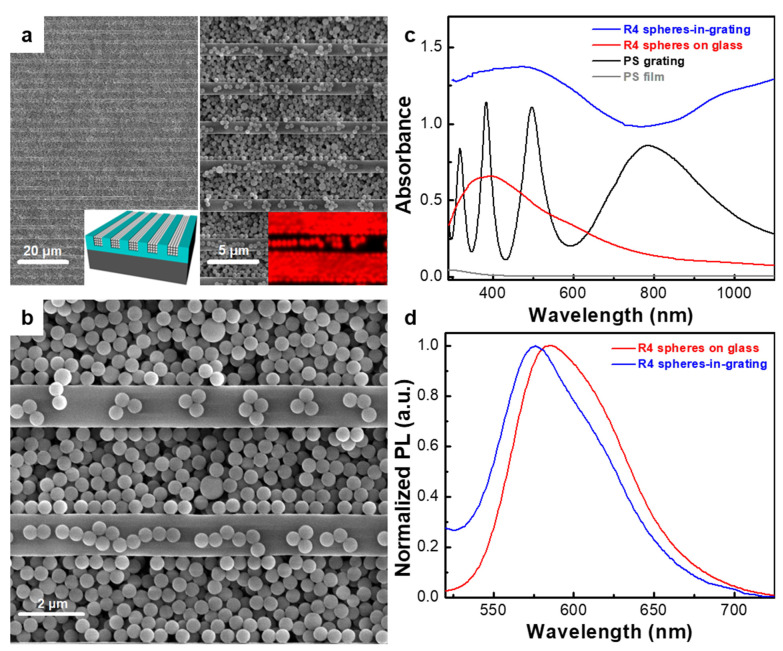
(**a**,**b**) SEM images of different magnification depicting Nile-red-dyed PS spheres (R4) in periodic PS grooves of a width of 2.5 µm and a depth of 2 µm. While the inset on the left schematically depicts the expected sphere-in-grating assembly, the inset on the right represents a fluorescence image of the obtained assembly. (**c**) Absorption spectra comparing an unpatterned PS film both with a PS grating before and after filling with R4 spheres, and with R4 spheres spin-cast on glass. (**d**) Normalized PL spectra exhibiting a blueshift from 583 nm to 575 nm when comparing R4 spheres spin-cast on glass to those incorporated in the grating. An excitation wavelength of 400 nm was used to record the PL spectra.

To fabricate spheres-in-grating assemblies with puzzling optoelectronic properties, we selected atactic PS (an optically transparent system) as the grating material and fluorescent spheres (PS particles stained with Nile red dye; R4) of an average diameter of 0.53 µm. By combining the embossing with CSA, we created periodic 2 µm deep PS grooves and filled them with R4 spheres (Figure 1a,b). The inset on the left of Figure 1a schematically depicts the expected four-layer structure. Indeed, as can be seen in Figure 1a–c, the overall incorporation of spheres in the grating was uniform over large areas and efficient, although the top first and sometimes second layers of spheres were less ordered than the bottom layers (Figure 1b), due to the rearrangements of these spheres that take place upon drying post-CSA, when the last remaining water evaporates.

In order to evaluate the optoelectronic properties of the aforementioned spheres-in-grating assembly, we performed absorption and photoluminescence (PL) measurements. Compared to layers of R4 spheres spin-cast on glass, the spheres-in-grating assembly exhibited an altered shape of the absorption spectrum, with increased intensity (Figure 1c), as the transmission of light became less efficient due to a change in the optical path length [6]. Then, by comparing the spheres-in-grating with an empty grating, one can observe that the absorbance maxima due to grating diffraction disappear upon sphere filling. These results indicate an overall good filling of the grating with R4 spheres, in agreement with the SEM observations. Additional PL measurements further revealed measurable differences in the emission spectra (Figure 1d). Spheres-in-grating assemblies based on R4 spheres exhibited a blueshifted emission from 583 nm to 575 nm, as compared to the spheres deposited on glass by spin-casting. This blueshift is most probably related to the spheres’ arrangement into quasi-ordered structures within the confined grating. According to the literature, such arrangements can modify the photonic density of states in the vicinity of spheres, leading to spectral modifications of the spontaneous emission from the fluorescent dye embedded in the PS spheres [49]. We do not exclude the possibility of this phenomenon being accompanied by another weak effect related to the degree of aggregation of dye molecules—the latter being known to induce changes in emission, including blueshift [50]. Dye-embedded fluorescent spheres adopting a spin-cast configuration on the substrate, represented by several or more layers of disordered but crowded spheres, can experience more contacts with the neighboring spheres as compared to their analogue spheres dispersed in quasi-ordered structures confined by the grating. This could slightly diminish the number of more aggregated conformations of dye molecules in the latter case and, thus, could further explain the observed blueshift. Another possible explanation for the observed PL shift related to the interaction of free fluorescent dye molecules with the polar glass surface was ruled out, as no changes in PL were observed for fluorescent spheres deposited on glass or on PS-covered glass substrates. The results presented in Figure 1 were further reconfirmed using spheres stained with Nile red fluorescent dye, but of a smaller diameter of only 0.25 µm (see Figure S1).

**Figure 2:**
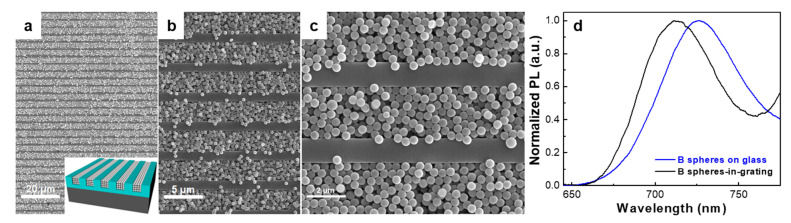
(**a**–**c**) SEM images of different magnification depicting PS spheres stained with fluorescent blue (**b**) dye and incorporated in periodic grooves of a width of 2.5 µm and a depth of 2 µm. The inset in (**a**) schematically depicts the expected sphere-in-grating assembly structure. (**d**) Normalized PL spectra illustrating a blueshift from 725 nm to 710 nm when comparing B spheres spin-cast on glass to those incorporated in the grating. An excitation wavelength of 410 nm was used.

In order to further explore the tunability of the optoelectronic properties characterizing the spheres-in-grating assemblies, we replaced the R4 spheres with blue-dyed PS spheres (B) of a similar diameter (0.49 µm). SEM images emphasizing the resulting assembly and its schematic structure are shown in Figure 2a–c. Again, the uniform and efficient filling over large-area samples was reconfirmed. In this case, B spheres also appeared rather disordered within the top two layers—an effect of post-CSA drying when the remaining water evaporates. Moreover, the emission spectra display an even stronger blueshift from 725 nm to 710 nm when comparing B spheres spin-cast on a glass substrate with those incorporated in the grating (Figure 2c). As stated above, the most plausible origin of the blueshift is related to the arrangement of fluorescent spheres into quasi-ordered structures within the confined grating.

**Figure 3:**
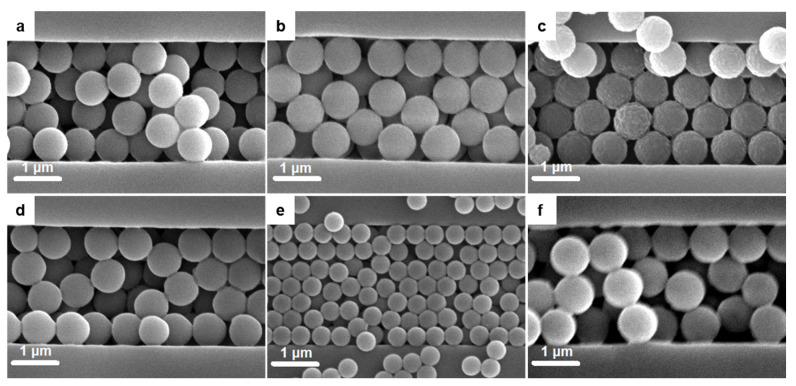
SEM images of spheres of different size stained with various dyes and assembled into 2.5 µm wide and 2 µm deep periodic grooves: (**a**) amino pink (AP), (**b**) amino red (AR), (**c**) yellow/Y, (**d**) jade green/G, (**e**) pink/P, and (**f**) Nile red (R1).

Moreover, we assembled other fluorescent spheres of different diameters that were stained with various dyes (including amino pink and amino red, yellow, green, etc.) into periodic grooves. The results are summarized in Figure 3 and Figure S2, and demonstrate the possibility of fast fabrication of large-area spheres-in-grating functional ordered/disordered hierarchical assemblies with altered emission properties. Here, the yellow-based spheres-in-grating assembly displayed a highly ordered hexagonal packing (Figure 3c), while amino red and Nile red spheres adopted a quasi-random, rather disordered packing (Figure 3b,d,f and Figure S2b,d,f). According to a geometric relationship diagram reported previously for three and four spheres along one of the close-packed directions, located within similar grooves [6], these two types of packing were expected to occur, as the corresponding ratio between the sphere diameter and the width of the groove (D/a) varied from ~0.32 (yellow spheres) to ~0.38 (amino red spheres). Instead, for seven spheres along one of the close-packed directions, located within a similar groove, only a disordered, random packing of spheres has been reported thus far [6]. In Figure 3e, we now demonstrate a (quasi-)ordered hexagonal packing of spheres that was achieved through the incorporation of spheres stained with pink dye (D/a~0.2) by utilizing the CSA method.

**Figure 4:**
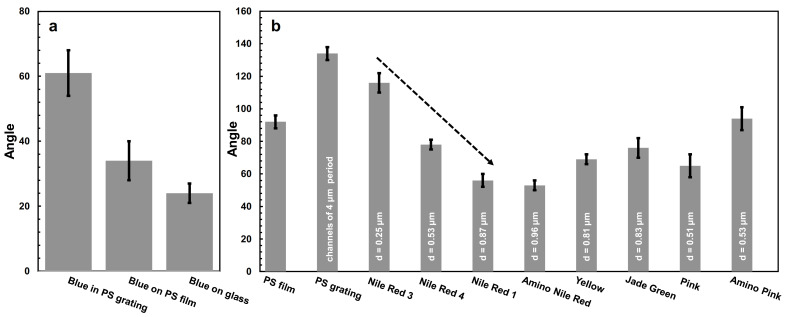
(**a**) Comparison of the contact angle displayed by surfaces fabricated by spin-casting the blue-dyed B spheres on a glass substrate and on a PS-covered glass substrate, and by their incorporation into a PS grating. (**b**) Contact angles measured for spheres of different diameter and fluorescent function incorporated into PS gratings, as compared to the contact angles of PS-covered glass and an empty PS grating.

In order to understand how the incorporation of spheres of different dimensions in the grating affects the wetting properties of the resulting structures (i.e., hydrophobicity vs. hydrophilicity), studies on static contact angle were further performed on all of the abovementioned samples. Firstly, we compared the contact angles of surfaces fabricated by spin-casting the blue-dyed B spheres both on a glass substrate and on a PS-covered glass substrate, and by their incorporation into the PS grating. The results presented in Figure 4a show a significant decrease in the hydrophilicity when covering the glass with PS prior the deposition of B spheres, and further when patterning the PS surface and filling the resulting grating with B spheres (the measured value of the contact angle increased from 24 ± 3 to 34 ± 6°, and more than doubled to 61 ± 7°, respectively; see Figure 4a). While the decrease in the glass’ hydrophilicity caused by its covering with a PS film is not surprising (assuming we are covering a cleaned glass displaying a contact angle < 10° [51] with a typical PS film exhibiting a contact angle of 92 ± 4° or so [52]), a further decrease in hydrophilicity by patterning the top PS film and filling the resulting grating with B spheres can be explained by a significant change in the surface roughness and texture [53,54] (see further the discussion of the effects of roughness on the contact angle in the next paragraph). The increase in hydrophobicity with patterning was obvious when comparing a PS film with an empty PS grating. As can be seen in Figure 4b, upon patterning, the contact angle value increased from 92 ± 4° to 134 ± 4°. This demonstrates that periodic grooves are capable of significantly altering the contact angle on PS without involving any modification of the surface chemistry.

Moreover, the contact angle measurements performed on all spheres-in-grating assemblies revealed that by adding spheres of different diameter and function to the grating, one can reduce the hydrophobicity of the latter (Figure 4b). Furthermore, the decrease in the contact angle seemed to be more significant when spheres of higher diameter were used. When analyzing the sequence of Nile red sphere systems incorporated in the PS gratings, we observed that with the increase in the sphere diameter, the contact angle decreased from 116 ± 6° (corresponding to R3, *D* = 0.25 µm), to 78 ± 3° (R4, *D* = 0.53 µm), to 56 ± 4° (R1, *D* = 0.87 µm; follow the dashed arrow in Figure 4b). While there are studies in the literature reporting that there is no effect of the particle size on the contact angle [55], it is agreed that the surface roughness causes changes in the hydrophobicity [56,57]. Moreover, there seems to be a different effect of roughness on the contact angle. On hydrophilic surfaces, roughness reduces the contact angle, while the opposite happens on hydrophobic surfaces [57]. In our case, we had a very rough hydrophobic PS grating surface that exhibited a contact angle of 134 ± 4°. A further filling of the grating with 0.25 µm Nile red spheres decreased the surface roughness and, thus, decreased the contact angle to 116 ± 6°. Although the 0.53 µm and 0.87 µm Nile red spheres exhibit a larger roughness (generally, an increase in the spheres’ diameter causes an increase in the surface texture and, thus, an increase in roughness), their contact angles are not larger, but smaller (78 ± 3° and 56 ± 4°, respectively), as we are now dealing with hydrophilic surfaces (i.e., the contact angle is <90°). Obviously, in this last situation, as an increase in roughness reduces the contact angle [57], surfaces incorporating larger and rougher spheres of 0.87 µm exhibit a lower contact angle.

Finally, the possibility of integrating the spheres-in-grating assemblies as critical components in various optoelectronic, sensing, photonic, or diagnosis devices, etc., often requires a further encapsulation of the obtained assemblies, e.g., deposition, on top of spheres-in-grating assemblies, of additional layers exhibiting various functions, including electrical or thermal conductivity, optical transparency, or a simple physical barrier to keep the spheres in the grating when, for instance, further processing in liquids is required, etc. Therefore, we developed a procedure to cover our spheres-in-grating assemblies with a protective layer of P(VDF-TrFE) polymer. We selected this polymeric system for its foreseen benefits in electronic [58] and piezoelectric [59] applications. The sequence of optical images provided in Figure S3 compares a PS grating before (Figure S3a) and after its filling (Figure S3b) with blue-dyed spheres with the same PS grating filled with B spheres, which was further “encapsulated”/covered using a 3 ± 1 µm thick layer of P(VDF-TrFE) (Figure S3c). The optical image shown in Figure S3c was taken by focusing the objective of the optical microscope through the layer of P(VDF-TrFE) located on top of the spheres-in-grating assembly, and revealed a good sample uniformity, confirming at the same time that the filling of the grooves with blue-dyed spheres remained unaffected upon encapsulation.

## 4. Conclusions

We successfully developed hierarchical ordered/disordered spheres-in-grating assemblies by using a combination of hot embossing and an adapted CSA method. These functional assemblies exhibited altered emission properties, as revealed by PL measurements, and displayed changes in wetting properties that depended not only on patterning, but also on the surface chemical function and dimensions of the incorporated spheres. Moreover, we encapsulated spheres-in-grating assemblies by successfully coating them with a few-micrometers-thick polymeric top layer, in order to demonstrate the potential of such assemblies as viable components in future optoelectronic, photonic, or medical devices.

## Data Availability

Data sharing not applicable.

## Supplementary Materials

The following supporting information can be downloaded at: https://www.mdpi.com/article/10.3390/nano12071084/s1. Table S1: Technical details of all fluorescent spheres used for incorporation in the PS gratings; Figure S1: (a–c) SEM images of different magnification depicting Nile Red dyed polystyrene spheres (R3) of an average diameter of 0.25 μm in periodic grooves of a width of 2.5 µm and a depth of 2 µm. (d) Normalized emission spectra exhibiting a blue-shift from 585 nm to 573 nm when comparing R3 spheres spin cast on glass to those incorporated in the grating. An excitation wavelength of 400 nm was used; Figure S2: SEM images of spheres of different size stained with various dyes and assembled into 2.5 µm wide and 2 µm deep periodic grooves: (a) Amino Pink (AP), (b) Amino Red (AR), (c) Yellow/Y, (d) Jade Green/G, (e) Pink/P and (f) Nile Red (R1); Figure S3: Optical images corresponding to an empty grating (a) and to a spheres-in-grating assembly made of blue-dyed spheres before (b) and after (c) encapsulation with a poly(vinylidene fluo-ride-co-trifluoroethylene)/P(VDF-TrFE)layer.
