# Supplementary material for: Spheres-in-Grating Assemblies with Altered Photoluminescence and Wetting Properties
Source: Nanomaterials (Basel). 2022 Mar 25;12(7):1084. doi: 10.3390/nano12071084 (PMC9000395; doi:10.3390/nano12071084)
Supplement: Supplementary file 1 [file nanomaterials-12-01084-s001.zip › nanomaterials-1636826-supplementary.pdf]

nanomaterials

Supplementary Materials

Spheres-In-Grating Assemblies with Altered Photoluminescence and Wetting Properties

Iuliana M. Handrea-Dragan [1,2], Adriana Vulpoi [1], Cosmin Farcău [1,3] and Ioan Botiz [1,*]

[1] Institute for Interdisciplinary Research in Bio-Nano-Sciences, Babes-Bolyai University, 400271 Cluj-Napoca, Romania; iuliana.dragan@ubbcluj.ro (I.M.H.-D.); adriana.vulpoi@ubbcluj.ro (A.V.); cfarcau@itim-cj.ro (C.F.)
[2] Faculty of Physics, Babes-Bolyai University, 400084 Cluj-Napoca, Romania
[3] National Institute for Research and Development of Isotopic and Molecular Technologies, 400293 Cluj-Napoca, Romania
* Correspondence: ioan.botiz@ubbcluj.ro

Table S1. Technical details of all fluorescent spheres used for incorporation in the PS gratings.

Type of nanoparticles	Name	Symbol	Diameter [μm]	Mean diameter [μm]
Fluorescent Particles	Nile Red	R3	0.1–0.3	0.25
	Nile Red	R4	0.4–0.6	0.53
	Nile Red	R1	0.7–0.9	0.87
	Amino Nile Red	AR	0.7–0.9	0.96
	Pink	P	0.4–0.6	0.51
	Amino Pink	AP	0.4–0.6	0.53
	Sky Blue	B	0.4–0.6	0.49
	Low Intensity Yellow	Y	0.7–0.9	0.81
	Jade Green	G	0.7–0.9	0.83

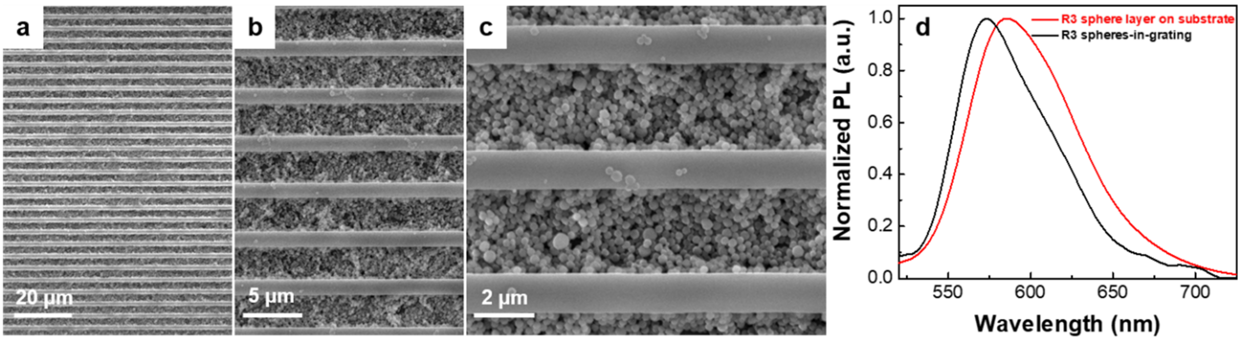

Figure S1. (**a**–**c**) SEM images of different magnification depicting Nile Red dyed polystyrene spheres (R3) of an average diameter of 0.25 μm in periodic grooves of a width of 2.5 μm and a depth of 2 μm. (**d**) Normalized emission spectra exhibiting a blue-shift from 585 nm to 573 nm when comparing R3 spheres spin cast on glass to those incorporated in the grating. An excitation wavelength of 400 nm was used.

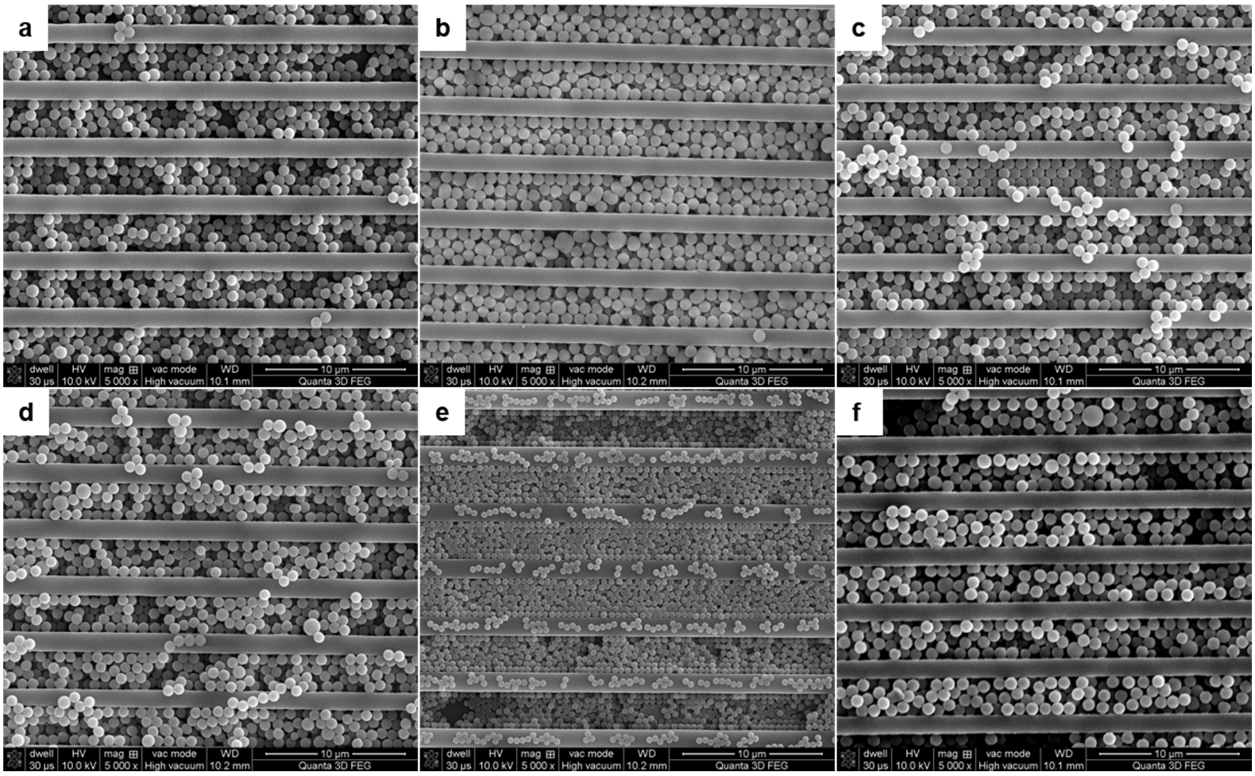

Figure S2. SEM images of spheres of different size stained with various dyes and assembled into 2.5 μm wide and 2 μm deep periodic grooves: (**a**) Amino Pink (AP), (**b**) Amino Red (AR), (**c**) Yellow/Y, (**d**) Jade Green/G, (**e**) Pink/P and (**f**) Nile Red (R1).

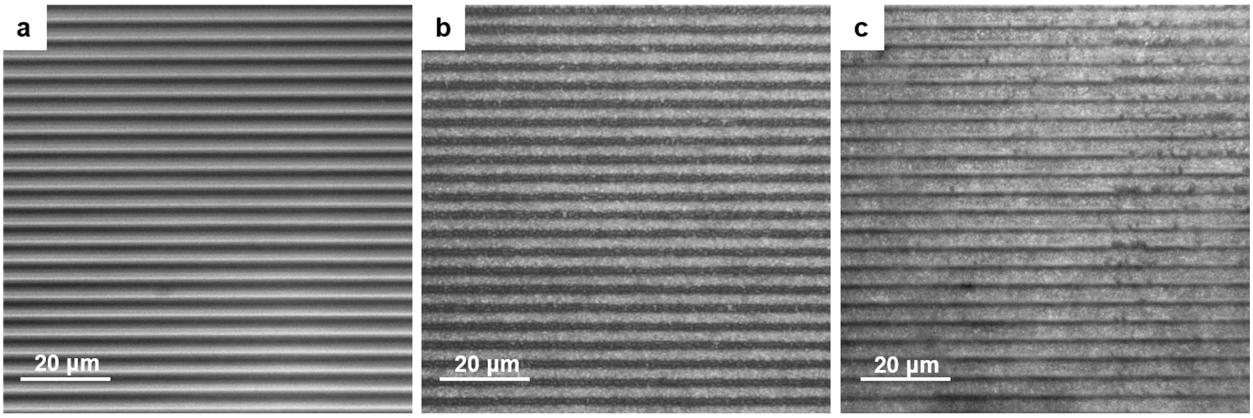

Figure S3. Optical images corresponding to an empty grating (**a**) and to a spheres-in-grating assembly made of blue-dyed spheres before (**b**) and after (**c**) encapsulation with a poly(vinylidene fluoride-co-trifluoroethylene)/P(VDF-TrFE)layer.
